# Flexible Peripheral Nerve Interfacing Electrode for Joint Position Control in Closed-Loop Neuromuscular Stimulation

**DOI:** 10.3390/mi15050594

**Published:** 2024-04-29

**Authors:** Sia Kim, Kang-Il Song

**Affiliations:** 1 Department of Biomedical Engineering, University of Ulsan, Ulsan 44610, Republic of Korea; 2 Division of Smart Healthcare, Pukyung National University, Busan 48513, Republic of Korea; 3 Digital Healthcare Research Center, Institute of Information Technology and Convergence, Pukyung National University, Busan 48513, Republic of Korea

**Keywords:** flexible neural interface, peripheral nerve, bi-directional neural interface, joint position control, neuromodulation

## Abstract

Addressing peripheral nerve disorders with electronic medicine poses significant challenges, especially in replicating the dynamic mechanical properties of nerves and understanding their functionality. In the field of electronic medicine, it is crucial to design a system that thoroughly understands the functions of the nervous system and ensures a stable interface with nervous tissue, facilitating autonomous neural adaptation. Herein, we present a novel neural interface platform that modulates the peripheral nervous system using flexible nerve electrodes and advanced neuromodulation techniques. Specifically, we have developed a surface-based inverse recruitment model for effective joint position control via direct electrical nerve stimulation. Utilizing barycentric coordinates, this model constructs a three-dimensional framework that accurately interpolates inverse isometric recruitment values across various joint positions, thereby enhancing control stability during stimulation. Experimental results from rabbit ankle joint control trials demonstrate our model’s effectiveness. In combination with a proportional–integral–derivative (PID) controller, it shows superior performance by achieving reduced settling time (less than 1.63 s), faster rising time (less than 0.39 s), and smaller steady-state error (less than 3 degrees) compared to the legacy model. Moreover, the model’s compatibility with recent advances in flexible interfacing technologies and its integration into a closed-loop controlled functional neuromuscular stimulation (FNS) system highlight its potential for precise neuroprosthetic applications in joint position control. This approach marks a significant advancement in the management of neurological disorders with advanced neuroprosthetic solutions.

## 1. Introduction

A peripheral nerve–electronics interface capable of restoring bidirectional communication in injured nerves can facilitate reliable control of prosthetic robots and aid in the diagnosis and therapy of neurological disorders. The safest and most reliable method to chronically interact with peripheral nerves may be achieved by combining mechanical modulus matching of nerve tissue with electrodes and employing minimally invasive procedures. As a promising solution for treating injured nervous systems, peripheral nerve cuff electrodes have been developed for neuroprosthetic applications in human studies. Specifically, spiral cuffs and flat interface nerve electrodes have proven effective in restoring its functions via neuromodulation. Functional neuromuscular stimulation (FNS) is an effective neuromodulation technique for alleviating motor function disorders caused by spinal cord injuries, strokes, and foot drops [1,2,3,4,5]. Recently, emerging neuromuscular stimulation techniques, such as optogenetic and ultrasound stimulations, have offered innovative strategies for targeted and non-invasive stimulation of specific neurons [6,7]. While these alternative methods mark considerable progress beyond the electrical stimulation techniques, they have yet to achieve their reliability and practicality for clinical application in treating and regenerating neuromuscular function as FNS [8].

To apply electrical stimulation to the FNS system, specific electrodes and stimulation methods should be equipped for adequate positioning in targeted stimulation regions. The percutaneously or intramuscularly implanted muscle stimulation method, which is a conventional method, has been used to target stimulation muscles to generate joint movements [9,10,11]. Although these approaches provide reliable results for joint movement control, they are limited in their application to high stimulation intensity and require electrodes to innervate the muscles to generate joint movements. As an alternative approach, direct nerve stimulation using a cuff electrode has provided the breakthrough in direct muscle stimulation, requiring either only a low stimulation intensity or fewer electrodes [12,13,14,15,16,17]. Intuitively, direct nerve and muscle stimulations have similarly induced neuromuscular junctions in muscles, and the optimal stimulation approaches for their intrinsic properties should be used for FNS applications.

In conventional joint position control, an understanding of the targeted limb/muscle should be used to create a control model that includes an inverse recruitment curve. When joint movements are generated by balancing contraction and relaxation in relevant agonist and antagonist muscles, the recruitment curves show different shapes owing to changes in the muscle length and velocity in each joint position [18]. A simple inverse recruitment curve has been used for joint position control using the FNS system, even though it may not fully reflect the targeted limb/muscle characteristics over the full range of motion. In addition to direct nerve stimulation, differences in intrinsic properties, such as the steep and narrow shape of inverse recruitment, could be considered in addition to the muscle and the nerve [19]. Consequently, an adequate recruitment model for direct nerve stimulation and its strategy are needed to represent changes in the variation of the recruitment curves generated for various joint positions for precise joint position control. Furthermore, in order to provide a neuromodulation strategy for recent biocompatible, flexible, and stretchable peripheral nerve interfacing electrodes [20,21,22], an adequate recruitment model is needed to fit the direct nerve stimulation approach.

The proposed neural interface platform, which includes flexible nerve electrodes and advanced neuromodulation techniques, provides a strategy for precise joint position control across the full range of motion, thereby paving the way for the application of flexible nerve electrodes. To achieve precise control performance, the proposed surface-based inverse recruitment model was constructed using a combination of individual recruitment curves and the barycentric coordination method. The performance of this model in controlling joint position was evaluated using a rabbit, by implementing the proposed method within a closed-loop FNS system and comparing it to a conventional recruitment curve approach. Our modeling approach was followed and demonstrated to be applicable for joint position control in a closed-loop controlled FNS system.

## 2. Materials and Methods

### 2.1. Modeling of Three-Dimensional Surface-Based Inversed Recruitment

The surface-based inverse recruitment model was designed based on the barycentric coordination method, with an inverse recruitment curve generated over the full range of motion in the ankle joint position. This curve was derived from the tetanic force generated by electrical stimulation trains applied to the tibial and peroneal nerves. The stimulation train was structured with a total duration of 20 s, including a stimulation period of 2 s and a rest period of 18 s. The pulse duration and frequency of the stimulation were set at 250 μs and 20 ms, respectively, and these parameters were modified based on the previous work [23]. Additionally, amplitude modulation was utilized in the stimulation train to support closed-loop joint position control, and the selected pulse duration and frequency were maintained consistently. Considering the range of motion in the rabbit hind limb, the maximum stimulation intensity was limited at 120 μA, adjusted in increments of 10 μA to mitigate muscle fatigue [24].

The measured tetanic forces were filtered using an average of 20 ms data windows. The filtered torques were then averaged and used as data points to generate recruitment curve surfaces. Ten stimulations were required to construct a recruitment curve for each joint. A decision surface was generated to determine the stimulation parameters; three-dimensional coordinates from the decision surface were used to define the ankle angle, torque, and stimulation levels. The stimulation parameters were determined using the barycentric coordinate equation of a plane. Three barycentric coordinate points were used to determine the stimulation amplitude level [24]. The overall procedure for this method is described as follows:

1. Given the point $\Gamma =(x_{d},y_{d})$ in a triangular plane, we can obtain the barycentric coordinates $\lambda_{1}$, $\lambda_{2}$, and $\lambda_{3}$ from their corresponding Cartesian coordinates.
2. We can express the Cartesian coordinates of point Γ in terms of the Cartesian components of the triangle vertices $x_{d}=\lambda_{1}x_{1}+\lambda_{2}x_{2}+\lambda_{3}x_{3}$ and $y_{d}=\lambda_{1}y_{1}+\lambda_{2}y_{2}+\lambda_{3}y_{3}$.
3. To determine the barycentric position of each $\lambda_{i}$, where i=1,2, or 3, we use the following equations:
  (1) $$\lambda_{1}=\frac{\left(y_{2}-y_{3}\right)\left(x_{d}-x_{3}\right)+(x_{3}-x_{2})(y_{d}-y_{3})}{\left(y_{2}-y_{3}\right)\left(x_{1}-x_{3}\right)+(x_{3}-x_{2})(y_{1}-y_{3})}$$
  (2) $$\lambda_{2}=\frac{\left(y_{3}-y_{1}\right)\left(x_{d}-x_{3}\right)+(x_{1}-x_{3})(y_{d}-y_{3})}{\left(y_{2}-y_{3}\right)\left(x_{1}-x_{3}\right)+(x_{3}-x_{2})(y_{1}-y_{3})}$$
  (3) $$\lambda_{3}=1-\lambda_{1}-\lambda_{2}$$
4. The point Γ lies inside the triangle if and only if $0<\lambda_{i}<1$, where i=1,2, or 3.
5. The input point Γ is selected from the nearest recruitment coordinate using the barycentric coordinate method.

### 2.2. Animal Preparation and Electrode Implantation

**Figure 1:**
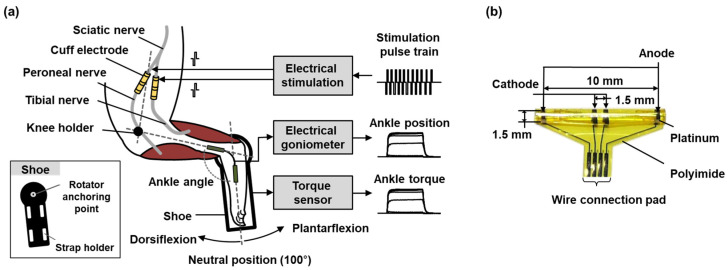
Animal experimental setup for three-dimensional surface-based inversed recruitment modelling (**a**) Schematic of animal experimental setup for measuring muscle force while electrical stimulation each on tibial and peroneal nerve at neutral joint position (100°), which is denoted dot lines. And the arrow line denoted the flow of the system. (**b**) Photograph of direct nerve stimulation electrode.

Animal experiments were performed using three 2–2.5 kg male New Zealand white rabbits. The animals were kept and handled in accordance with the regulations of the Institutional Animal Care and Use Committee of the Korea Institute of Science and Technology (approval number: KIST-2019-111). For nerve cuff electrode implantation, recruitment modeling, and joint position control in vivo, the animal was anesthetized with a Zoletil- Rompun cocktail (1 mL/kg, intramuscular injection; Zoletil, Virbac, NSW, Australia; Rompun, Bayer Korea, Republic of Korea). The animal experimental procedure has been modified in our previous study [12]. The cuff electrode was implanted through an incision in the thigh, from the vertebral column to the knee. The biceps femoris and semitendinosus were identified and retracted to expose the tibial and peroneal nerves above the branch from the sciatic nerve, and the electrode was positioned on each nerve (Figure 1a). Considering the sciatic nerve dimensions, the cuff electrode was fabricated by depositing platinum on a thin polyimide film substrate, and it consisted of a rectangular electrode (0.25 mm wide, 7 mm long) with a self-curling shape (Figure 1b). For electrical stimulation, a pair of two-center platinum ring electrodes was used as the cathode, and two other pairs of outer electrodes were used as the anode. Each pair of electrodes was connected to an electrical stimulator (AM 2200, A-M Systems, WA, USA) through medical wires (CZ 1209, Cooner Wire, CA, USA). Furthermore, we applied a flexible electrode in this study to demonstrate its applicability to recent peripheral nerve interfaces, where electrodes were used in our previous work [20].

### 2.3. Data Acquisition and Neuromodulation

To modify the three-dimensional recruitments, the electrical stimulation-induced muscle force was measured using a torque transducer with an apparatus system. The apparatus system was used to measure the muscle force holding each joint in the dorsiflexion–plantarflexion position. Throughout the experiments, the rabbits were placed on the left side above the designed apparatus. The knees of the rabbits were secured using a pair of concave cups to prevent knee rotation. The hip and knee angles (i.e., the angles between the tibia and femur and between the femur and spine) were set to 65° and 83°, respectively. In addition, the right foot was secured on a shoe to measure the torque (Figure 1a) using a torque transducer (QWFK-8M, Honeywell, NC, USA) coupled to the shoe shaft. The torque data were digitalized using an analog-to-digital converter board (PXI-6143, National Instruments, AU, USA) with a 16-bit resolution and a sampling rate of 25 kHz. To elicit the torques generated during dorsiflexion and plantarflexion, a charge-balanced biphasic current pulse train was induced on each peroneal and tibial nerve and generated by an electrical stimulator with a programmed pulse generator, which used a 16-bit-resolution digital-to-analog converter board (PXI-6733, National Instruments, USA).

### 2.4. Joint Position Control Setup

**Figure 2:**
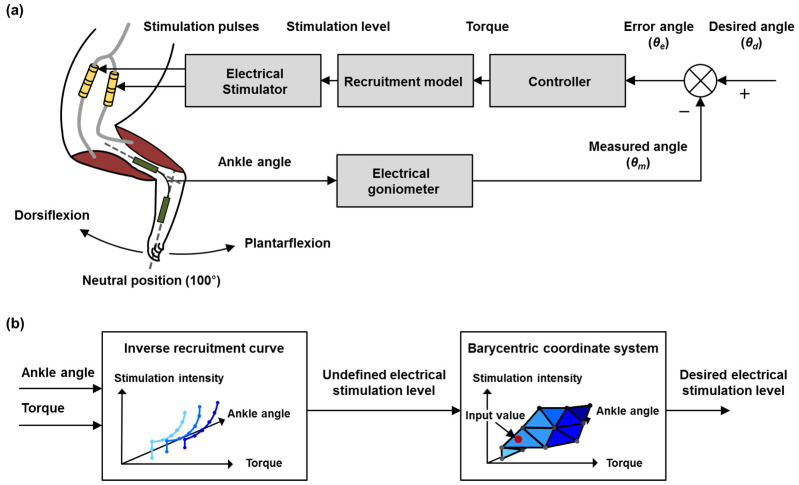
Joint position control experiment using proposed three-dimensional recruitment model. (**a**) Schematics of joint position control for evaluating the performance of three-dimensional recruitment model. (**b**) Block diagram of surface-based inverse recruitment model applied on the closed-loop FNS system control.

To demonstrate the feasibility of the joint position control produced using surface-based inverse recruitment, the ankle position was controlled while the surface-based inverse recruitment was applied to a closed-loop FNS system composed of a stimulation-cuff electrode, an electrical stimulator, the surface-based inverse recruitment, a proportional–integral–derivative (PID) controller, and a goniometer (Figure 2a). To control the joint position, the measured ankle angle was fed to the PID controller as a feedback signal. The stimulation level was then adjusted to minimize the error between the desired and measured angles. The cuff electrode was positioned on both the tibial and peroneal nerves to elicit ankle angle movements using an electrical stimulation train. The implemented stimulation electrode and the other experimental apparatus were identically positioned, as described in Section 2.1 and Section 2.2. The PID controller calculated the muscle force from the error angle by comparing the differences between the desired and measured angles obtained from the goniometer. The calculated torque was then converted into a stimulation level using a surface-based inverse recruitment curve. The calculated stimulation level was used to modulate the amplitude of the stimulation pulse train through an electrical stimulator to elicit the ankle position. The PID controller is defined as the vector *u*(*t*) as:

(4) $$u\left(t\right)=u\left(t-1\right)+K_{p}\left(\theta_{e}\left(t\right)-\theta_{e}\left(t-1\right)\right)+K_{i}\left(\frac{\theta_{e}\left(t\right)-\theta_{e}\left(t-1\right)}{2}\right)+K_{d}\left(\theta_{e}\left(t\right)-2\theta_{e}\left(t-1\right)+\theta_{e}\left(t-2\right)\right)$$

where u(t) is the output vector representing the torque at time t. Error-angle vector $\theta_{e}(t)$ is defined as $\theta_{e}\left(t\right)=\theta_{d}\left(t\right)-\theta_{m}\left(t\right)$, where $\theta_{d}\left(t\right)$ and $\theta_{m}\left(t\right)$ represent the desired and measured joint-angle vectors at time t, respectively (Figure 2b). $\theta_{e}\left(t-1\right)$ and $\theta_{e}\left(t-2\right)$ are defined by the time delay of the error term. The PID gains were manually tuned based on the Ziegler–Nichols method [25], and the controller period was set to 20 ms to account for the repetitive stimulation frequency. In addition, the surface-based inverse recruitment model was implemented on the PID controller for application to the closed-loop FNS system (Figure 2b). All systems were implemented in LabVIEW (National Instruments, AU, USA).

### 2.5. Joint Position Performance Evaluation

To evaluate the performance of the ankle-position control developed through surface-based inverse recruitment, its performance was compared with the performance of that developed using the inverse isometric recruitment curve that was applied to the closed-loop FNS system under identical experimental conditions. The performance of the ankle-position control was evaluated according to the steady-step responses measured at ankle angles of 70°, 90°, 110°, and 130° as the desired trajectories. The performance requirements for the controllers were set as follows: Overshoots < 10%, rising time < 0.5 s, settling time < 1.5 s, and steady-state error between −5% and 5% for a step input at the desired ankle angle, where overshoot is defined as the rate by which the peak response exceeded the desired one. The rising time is defined as the time required for the step response to increase from 10% to 90%. The settling time is defined as the time required for the step response to decrease to and remain within 5% of its final value. In addition, the integral time-weighted absolute error (*ITAE*) was calculated to evaluate the position control performance. The *ITAE* is defined as follows:

(5) $$ITAE=\int_{0}^{T}t\left|e\left(t\right)\right|dt,$$

where t is the control time, and e(t) is the difference between the desired and measured ankle positions observed throughout the controller.

The statistical analysis was performed Statistical analysis was performed using one-way ANOVA with Tukey’s multiple comparison test with Origin 2020 software.

## 3. Results

### 3.1. Estimation of Surface-Based Inverse Recruitment Model

**Figure 3:**
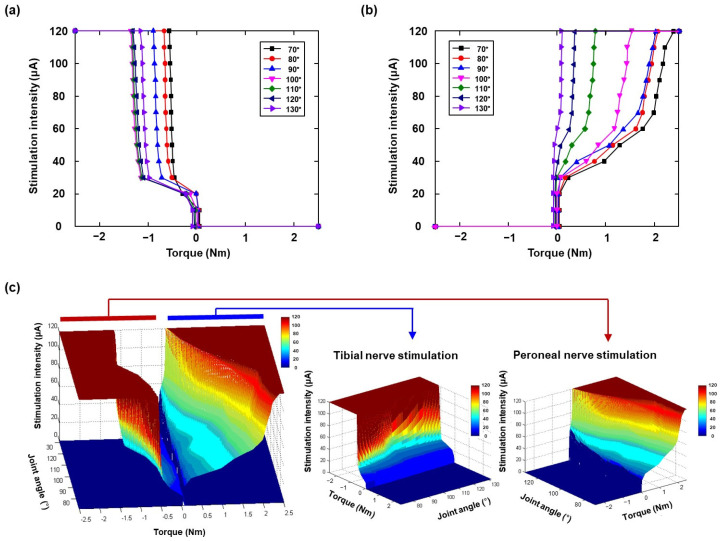
The inverse recruitment curves at different initial joint position (70°, 80°, 90°, 100°, 110°, 120°, and 130°), respectively: inverse recruitment for (**a**) tibial nerve stimulation, (**b**) peroneal nerve stimulation. And (**c**) three-dimensional decision planes obtained for full range of motion, tibial nerve stimulation, and peroneal nerve stimulation.

The visualized surface-based inverse recruitment model is shown in Figure 3. To construct the model, the electrical stimulation-induced isometric muscle force was measured at different initial ankle angle positions with each stimulation site of the tibial and peroneal nerves (Figure 3a,b). In general, the inverse recruitment curves showed a monotonic increase in each initial joint position while increasing the stimulation intensities and muscle force. Specifically, for the recruitment curve at an ankle angle of 70°, a steep slope was observed in peroneal nerve stimulation (Figure 3a,b). In addition, the slopes decreased with increasing initial joint positions (Figure 3b). As the initial ankle angle increased, the slope of the inverse recruitment curve became steeper in the tibial nerve plot, where the maximum torque decreased. In addition, in the peroneal nerve stimulation, the slope of the inverse isometric recruitment curve became less steep with increasing initial ankle angle, and the maximum torque increased. These results were also observed in other rabbit experiments. In the experiments, the neutral position was set to 100°. To decrease the initial angle successively in the range 100–80°, the center of the inverse isometric recruitment curve must be shifted leftward; however, the ankle angles increased step-by-step from 100° to 130°, and the center of the inverse isometric recruitment curve shifted leftward. The surface-based inverse recruitment curve for the full range of motion is shown in Figure 3c, which is composed of the recruitment curves for the tibial and peroneal nerves.

### 3.2. Closed-Loop Ankle-Position Control via Surface-Based Inverse Recruitment Model

**Figure 4:**
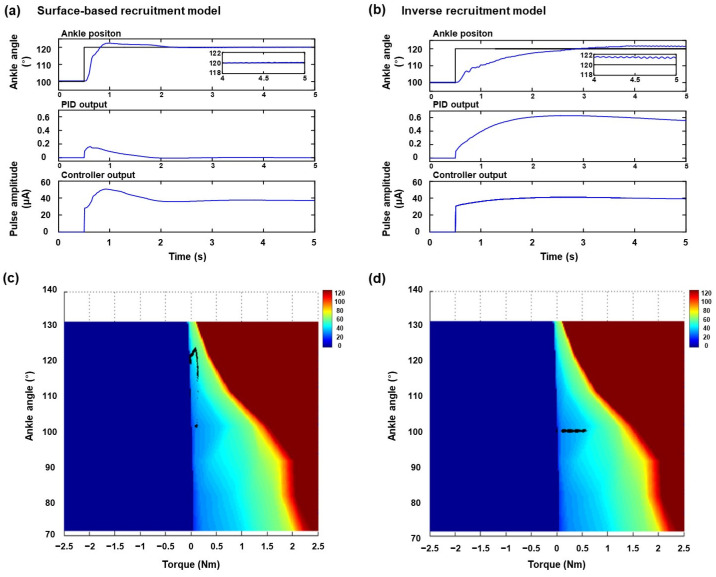
Representative results of ankle position control in plantarflexion at 120°: steady-state responses of (**a**) surface-based inverse recruitment model and (**b**) inverse isometric recruitment curve, and corresponding controller trajectories output during ankle-position control in (**c**) surface-based inverse recruitment model and (**d**) inverse isometric recruitment curve.

**Figure 5:**
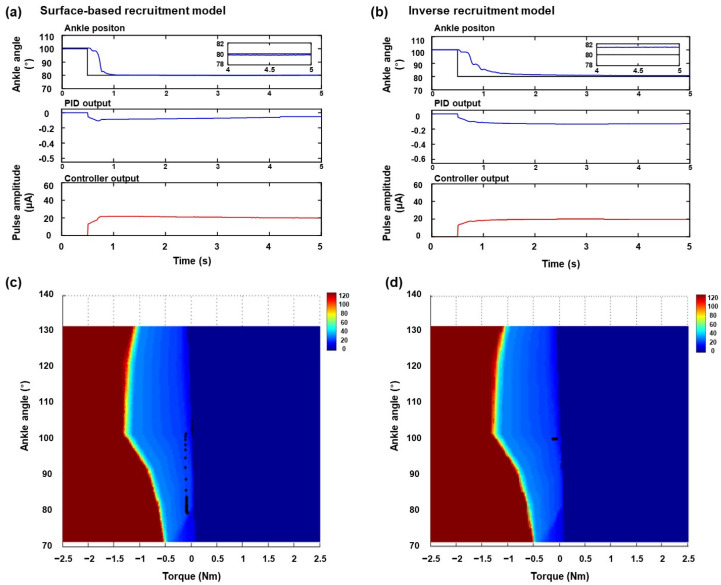
Representative results of ankle position control in dorsiflexion at 80°: steady-state responses of (**a**) surface-based inverse recruitment model and (**b**) inverse isometric recruitment curve, and corresponding controller trajectories output during ankle-position control in (**c**) surface-based inverse recruitment model and (**d**) inverse isometric recruitment curve.

**Figure 6:**
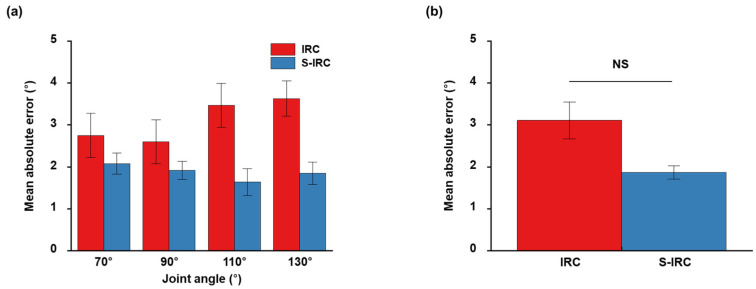
Mean absolute errors of ankle-position control: (**a**) mean absolute errors were evaluated by different inverse recruitment methods at different ankle positions (70°, 90°, 110°, and 130°), and (**b**) average of mean absolute error in every joint angle position at inverse recruitment and surface inverse recruitment curve, respectively. Statistical analysis was performed using one-way ANOVA with Tukey’s multiple comparison test (NS (not significant) *p* > 0.05).

To demonstrate the performance of the surface-based inverse recruitment model in the closed-loop ankle position, the joint position was controlled in each dorsiflexion and plantarflexion position. The representative results of ankle position control at the plantarflexion position of 120° are shown in Figure 4. Figure 4a,b shows the results of ankle position control under a steady-state response using surface-based inverse recruitment and the inverse isometric recruitment curve, respectively. To compare with these recruitment curves, the ankle position, PID output, and amplitude command were displayed; the black and blue lines denote the desired ankle angle and actual ankle position, respectively. The controlled angle position showed a faster rising time, shorter settling time, and lower steady-state error than the conventional inverse recruitment curve, when the surface-based inverse recruitment model was used for joint position control. In the PID-output subplot, the amplitude in the surface-based inverse recruitment model was smaller than that in the inverse isometric recruitment curve. In the amplitude-command subplot, the pulse amplitude in the surface-based inverse recruitment model was higher than that in the inverse isometric recruitment curve within 2 s. Additionally, the electrical stimulation amplitude showed a shape similar to that of the inverse recruitment model. To demonstrate the effectiveness of joint position control, the controller trajectory outputs were overlapped on the surface-based inverse recruitment plane, where the black dot denotes the PID controller output (Figure 4c,d). During the ankle-position control, the controller trajectory output followed the surfaces of the different ankle angles generated using the surface-based recruitment model. Figure 5 shows the results obtained for the ankle position at the dorsiflexion position of 80°. Overall, the ankle position control results were shown in both the surface-based recruitment model and the conventional recruitment curve. However, short rising time and settling time, and low steady-state error were obtained in the proposed model (Figure 5a). Furthermore, the surface-based isometric model showed a smaller steady-state error than the inverse recruitment curve. The controller trajectory output followed the surface-based inverse recruitment model during the ankle position control (Figure 5c). The results of the performance of the ankle position control evaluated for the different recruitment methods at different desired ankle positions are listed in Table 1. Overall, the joint position control in the plantarflexion position (70° and 90°) showed less overshoot, fast rising time and settling time, and ITAE than the dorsiflexion position (110° and 130°). Among these, the proposed model showed less overshoot, fast rising time and settling time, and ITAE than the conventional inverse recruitment curves. Finally, the mean absolute errors of ankle position control were calculated to verify their applicability to other animals (Figure 6). The root mean absolute error (RME) values at different joint positions were 2.75° ± 0.53°, 2.60° ± 0.52°, 3.47° ± 0.52°, and 3.63° ± 0.42°, respectively (Figure 6a), and the data are represented as the mean ± S.D. At the inverse recruitment curve and the surface-based inverse recruitment curve, the average RME was 3.11° ± 0.44 and 1.87° ± 0.16 at the inverse recruitment curve and the surface-based inverse recruitment curve, respectively (Figure 6b).

## 4. Discussion

In this study, a surface-based inverse recruitment model was proposed to control the joint position in full range of motion for a flexible peripheral interface-based closed-loop FNS system. Previous studies have shown that direct nerve stimulation is applicable in the FNS to generate limb movements and modulate its functions using low stimulation intensities [14,15,26]. Technically, this low stimulation intensity has the benefit of developing an FNS system for miniaturization and full implantation systems. However, the limitation of control stability from the steep slope of stimulation intensity versus joint movements (i.e., recruitment curve) remains an issue for precise joint position control. Our proposed surface-based inverse recruitment model compensates for this limitation during joint position control by providing a decision surface using a barycentric coordination method. Furthermore, we provided a neuromodulation strategy for recent flexible and stretchable peripheral interfacing to implement the FNS system. The overall concept of the proposed model is that the decision plane was extended from the curve to the surface to compensate for the intrinsic properties of each joint position (Figure 2b). As shown in Figure 4c and Figure 5c, the controller trajectories were followed on the decision surface of the recruitment model to adjust the characteristics of each joint position. The results prove that the surface decision plane was compensated for by the lower controllability of the steep recruitment slopes in direct nerve stimulation. In addition, the data listed in Table 1 prove that our proposed model shows a faster rising time, shorter settling time, and smaller steady-state error than the conventional inverse isometric recruitment curve.

In the surface-based inverse recruitment model, the measured muscle force was influenced by varying ankle positions. Durfee [23] demonstrated that the relationship between the muscle force and stimulation intensity monotonically increased with increasing ankle angle in the dead, linear, and saturation zones; thus, the linear zone determined the overall performance of the joint position control. Crago [18] observed that recruitment characteristics were nonlinear and varied depending on the stimulators and muscle length. The surface-based inverse recruitment model, constructed from inverse recruitment data at different joint positions, accounted for variation in muscle characteristics and improved control in regions where the recruitment curve’s steepness reduced controllability. Muscle force variation was measured by direct nerve stimulation with recorded isometric force (Figure 3). As described in Section 3.1, the isometric muscle force incrementally increased with higher electrical stimulation intensity. Additionally, the tibial nerve plot demonstrated stronger muscle force compared to the peroneal nerve plot. To estimate the surface-based inverse recruitment model, the barycentric method [24] was employed using the three nearest data points surrounding the desired output in the joint-angle coordinates, which are locally linear even though the entire system is globally nonlinear. However, a fused tetanic force emerged in the plots of both the tibial and peroneal nerves at a current intensity of 30 µA. Notably, the plot for the peroneal nerve in particular exhibits fast muscle contraction within 1 s at the onset of the stimulation pulse train and during 2 s stimulation-pulse periods. Therefore, if a sufficient number of steady-state data points are mapped closely in the desired output space, an inverse recruitment model can be established through linear interpolation, resulting in minimal steady-state output error.

The improved ankle-position control performance achieved using the surface-based inverse recruitment model in the closed-loop FNS is shown in Figure 6 and detailed in Table 1. To evaluate the performance of this model, both the surface-based inverse recruitment model and the inverse isometric recruitment curve were implemented within a closed-loop FNS system. This setup demonstrated the adjustability of the proposed model for controlling ankle position. A conventional PID controller was employed to regulate the FNS system, effusively mitigating disturbances and parameter variations [27]. In this study, the discrete-velocity-form PID controller was selected to compensate for the nonlinearity of the neuromuscular system and prevent reset windup.

Following the results obtained using the PID controller, the proposed surface-based inverse recruitment adjusted the stimulation level to the proper position. The output of the PID controller adjusted the amplitude of the electrical stimulation to elicit the targeted joint angle. The overall control performance of the model exhibited a fast-rising time and a short settling time. A comparison of the steady-state errors in the position controls between the isometric recruitment curve and the surface-based model revealed that the latter maintained a lower error rate, under 1°. Notably, Figure 4a demonstrates that the steady-state error in the inverse recruitment curve begins oscillating after only 4 s, increasing with longer durations of ankle position control. Hence, the surface-based inverse recruitment model is more stable and better resists oscillation in steady-state error as it adapts to muscle characteristics across various ankle positions.

During joint position control under conditions of plantar flexion and dorsiflexion, they exhibited some overshoot in the plantarflexion position, including a surface-based inverse recruitment model. Despite this, the model’s performance was significantly enhanced, as evidenced by its rapid rising time, short settling time, and minimal steady-state error. Additionally, we calculated the ITAE to compare performances between models under both plantarflexion and dorsiflexion. The surface-based model and the inverse recruitment curve recorded ITAE values of 8.54 and 24.88 under plantarflexion at 120°, respectively, and 11.35 and 14.93 under dorsiflexion at 80°, respectively. Thus, the proposed model exhibited superior performance under dorsiflexion.

The enhanced control performance of the surface-based inverse recruitment model also indicates that the decision plane required to select the stimulation level in the controller aligns with the surface of the recruitment model, accommodating variations in ankle positions as depicted in Figure 4b and Figure 5b. Consequently, this model effectively compensates for the muscle characteristics observed at each joint position, optimizing control outcomes.

Furthermore, this study provides a neuromodulation strategy for implementing a flexible peripheral interface in a closed-loop FNS system. Our previous research confirmed that the charge delivery density was 0.382 mC/cm^2^ for flexible/stretchable cuff electrodes [23]. In this study, we utilized the flexible Pi film electrode, which has a charge delivery density of 0.315 mC/cm^2^, and successfully demonstrated stable joint angle control. Although the material properties of flexible Pi film slightly differ from those of recent stretchable [23] and variable flexible cuff electrodes [17,26,28,29], these properties primarily affect biocompatibility and reliability concerning organ interactions, rather than electrical stimulation. We believe that our demonstration offers promising applications for flexible/stretchable electrode-based FNS systems in neurorehabilitation and could also offer supportive solutions in human-robotics and brain–machine interfaces.

## 5. Conclusions

In summary, we have shown that surface-based inverse recruitment is a suitable method for controlling ankle position through nerve stimulation. The proposed model compensates for variations in, as well as the steep slope of, the recruitment curve under the full range of motion by extending the surface from the inverse isometric recruitment curve; this demonstrates its potential as an alternative method of position control in a closed-loop FNS system. Furthermore, the proposed method could be applied to an FNS system to control ankle position feedback by recording neural signals and electrically stimulating the nerves.

In conclusion, this study proposes a surface-based method to determine the stimulation parameters required for electrical nerve stimulation within a closed-loop FNS system. This method provides an efficient approach for controlling complex body movements by considering limb characteristics throughout the full range of motion during electrical stimulation of nerves. We believe that it provides a viable solution for precise joint control, even in the clinical field. Additionally, we anticipate its application in realistic human prosthetic technologies, such as human augmentation, in conjunction with advanced bio-integrated electronic technologies. In animal experiments, the joint position control performance demonstrated a faster step response and lower steady-state error compared to conventional recruitment models when both were accessed using a PID controller. In future studies, this proposed method could be implemented in a fully implanted feedback-controlled FNS system, enabling functional control of limb motion during activities such as slow walking and balancing weight while standing.

## Figures and Tables

**Table 1:** Performance of ankle-position control evaluated by different inverse recruitment methods at different ankle positions.

Inverse Recruitment Method	Joint Angle (°)	Performance Indices			
		Overshoot (%)	Rising Time (s)	Settling Time (s)	ITAE (Degree)
Inverse isometric recruitment curve	70	0	1.07	2.23	15.84
	90	0	1.12	2.16	14.15
	110	0	1.75	1.97	24.13
	130	0	1.7	1.98	25.41
Surface-based inverse recruitment model	70	0	0.36	0.43	12.11
	90	0	0.39	0.47	10.95
	110	2.5	0.25	1.57	8.21
	130	2.8	0.21	1.63	9.47

## Data Availability

The original contributions presented in the study are included in the article, further inquiries can be directed to the corresponding author.
